# Supplementary material for: EnzML: multi-label prediction of enzyme classes using InterPro signatures
Source: BMC Bioinformatics. 2012 Apr 25;13:61. doi: 10.1186/1471-2105-13-61 (PMC3483700; doi:10.1186/1471-2105-13-61)
Supplement: Addtional file 5 — The Java code to format the data files, evaluate and predict. The file enzml_java_code.tar.gz contains the Java code used to format database data to ARFF and XML formats, to execute cross and train-test (jackknife) evaluations and to record evaluation results to database. More information is included in the readme.txt file and the Javadoc files. The code can be used with a MySQL database. To use a different database software, other JDBC drivers might be required. [file 1471-2105-13-61-S5.gz › java_code/enzml2011/doc/uk/ac/ed/inf/enzml/test/mulan/attributesfilter/AttributesFilterDataTest.html]

AttributesFilterDataTest


---


|  |  |  |
| --- | --- | --- |
| **Overview** \| **Package** \| **Class** \| **Use** \| **Tree** \| **Deprecated** \| **Index** \| **Help** | |  |
| **PREV CLASS**   **NEXT CLASS** | **FRAMES**    **NO FRAMES**     **All Classes** |  |
| SUMMARY: NESTED \| FIELD \| CONSTR \| METHOD | DETAIL: FIELD \| CONSTR \| METHOD |  |


---


## uk.ac.ed.inf.enzml.test.mulan.attributesfilter Class AttributesFilterDataTest

```
java.lang.Object
  junit.framework.Assert
      junit.framework.TestCase
          uk.ac.ed.inf.enzml.test.weka.DataTableTest
              uk.ac.ed.inf.enzml.test.mulan.attributesfilter.AttributesFilterDataTest
```

**All Implemented Interfaces:**: junit.framework.Test

---

``` public class AttributesFilterDataTest extends DataTableTest ```

---

**Field Summary**

**Fields inherited from class uk.ac.ed.inf.enzml.test.weka.DataTableTest**

`ATTRIBUTE_FIELD, CLASS_FIELD, INSTANCE_FIELD`


**Constructor Summary**

`AttributesFilterDataTest()`


| **Method Summary** | |
| --- | --- |
| `java.lang.String` | `getInsertData()` |
| `void` | `setTableName()` |
| `void` | `testTableContent()` |

**Methods inherited from class uk.ac.ed.inf.enzml.test.weka.DataTableTest**

`createTestTable, getTableName, getTableReader, initialise, setTableName, testLoadData, testTableInstanceAttribute, testTableInstanceClass`

**Methods inherited from class junit.framework.TestCase**

`countTestCases, getName, run, run, runBare, setName, toString`

**Methods inherited from class junit.framework.Assert**

`assertEquals, assertEquals, assertEquals, assertEquals, assertEquals, assertEquals, assertEquals, assertEquals, assertEquals, assertEquals, assertEquals, assertEquals, assertEquals, assertEquals, assertEquals, assertEquals, assertEquals, assertEquals, assertEquals, assertEquals, assertFalse, assertFalse, assertNotNull, assertNotNull, assertNotSame, assertNotSame, assertNull, assertNull, assertSame, assertSame, assertTrue, assertTrue, fail, fail, failNotEquals, failNotSame, failSame, format`

**Methods inherited from class java.lang.Object**

`equals, getClass, hashCode, notify, notifyAll, wait, wait, wait`

**Constructor Detail**

### AttributesFilterDataTest

```
public AttributesFilterDataTest()
```


**Method Detail**

### getInsertData

```
public java.lang.String getInsertData()
```

:   **Overrides:**: `getInsertData` in class `DataTableTest`

---


### setTableName

```
public void setTableName()
```

---


### testTableContent

```
public void testTableContent()
```


---


|  |  |  |
| --- | --- | --- |
| **Overview** \| **Package** \| **Class** \| **Use** \| **Tree** \| **Deprecated** \| **Index** \| **Help** | |  |
| **PREV CLASS**   **NEXT CLASS** | **FRAMES**    **NO FRAMES**     **All Classes** |  |
| SUMMARY: NESTED \| FIELD \| CONSTR \| METHOD | DETAIL: FIELD \| CONSTR \| METHOD |  |


---
